# Comparison of Next-Generation Sequencing and Fluorescence In Situ Hybridization for Detection of Segmental Chromosomal Aberrations in Neuroblastoma

**DOI:** 10.3390/diagnostics11091702

**Published:** 2021-09-17

**Authors:** Eojin Kim, Boram Lee, Ji Won Lee, Ki Woong Sung, Jung-Sun Kim

**Affiliations:** 1 Department of Pathology and Translational Genomics, Samsung Medical Center, Sungkyunkwan University School of Medicine, Seoul 06351, Korea; mooncalf35@naver.com (E.K.); boram.lee@samsung.com (B.L.); 2 Samsung Genome Institute, Research Institute for Future Medicine, Samsung Medical Center, Seoul 06351, Korea; 3 Department of Pediatrics, Samsung Medical Center, Sungkyunkwan University School of Medicine, Seoul 06351, Korea; kiwoong.sung@samsung.com; 4 Department of Health Sciences and Technology, Samsung Advanced Institute for Health Sciences and Technology, Sungkyunkwan University, Seoul 06351, Korea

**Keywords:** neuroblastoma, next-generation sequencing (NGS), fluorescence in situ hybridization (FISH), segmental chromosomal aberration (SCA), 1p deletion, 11q deletion, 17q gain

## Abstract

The aim of this study was to compare next-generation sequencing (NGS) with the traditional fluorescence in situ hybridization (FISH) for detecting segmental chromosomal aberrations (SCAs) such as 1p deletion, 11q deletion and 17q gain, which are well-known predictive markers for adverse outcome in neuroblastoma. The tumor tissue obtained from 35 patients with neuroblastoma was tested by FISH and targeted NGS, which is specially designed to detect copy number alterations across the entire chromosomal region in addition to mutations in 353 cancer-related genes. All chromosomal copy number alterations were analyzed using the copy number variation plot derived from targeted NGS. FISH was performed to detect 1p deletion, 11q deletion and 17q gain. The copy numbers of 1p, 11q, and 17q obtained via NGS were correlated with those acquired via FISH. The SCAs determined by NGS were matched with those by FISH. Most 17q gain of mismatched cases detected by NGS alone showed a subsegmental gain of 17q. FISH revealed 11q deletion and 17q gain in a few tumor cells of two cases, which were not detected by NGS. NGS can be a sensitive complementary and alternative method to the conventional FISH for detecting SCAs.

## 1. Introduction

Neuroblastoma (NB) is a pediatric solid tumor and the most common extracranial neoplasm among children aged 1–14 years [1]. It is derived from the neural crest precursor cells of the sympathetic nervous system. The clinical behavior varies remarkably according to the histologic subtype of tumor, age, Mitosis-Karyorrhexis Index (MKI), serum and urine parameters, and molecular status of the tumor [2,3]. One of the molecular factors, segmental chromosomal aberration (SCA) including 1p deletion, 11q deletion, and 17q gain, has been associated with poor outcome [4,5,6]. Accordingly, patients diagnosed with NB should be accurately stratified depending on genetic or molecular features to improve treatment and prognosis.

Fluorescence in situ hybridization (FISH) is the conventional method used to detect SCAs in NB. FISH has been an important tool in the molecular diagnosis of various chromosomal abnormalities, including gain, deletions, and translocations, which are detecting by locus-specific probes targeting at specific DNA sequences. A limited number of these fluorescently labeled probes can be used at one time [7]. However, the advent of next-generation sequencing (NGS) technique has opened up new possibilities. NGS is a high-throughput method, allowing massive DNA sequencing in parallel [8]. In addition to whole genome, whole exome and whole transcriptome approaches, targeted NGS sequencing of a set of clinically meaningful genes has been used more practically for clinical applications [9]. NGS has been applied to detect single nucleotide variations and copy number variations of multiple genes as well as gene fusions, which play a significant role in diagnosis, prediction of outcome, and potential targeted cancer therapy of solid tumors [10]. Further, the inclusion of numerous sets of probes to detect genes through whole chromosomes provides another potential tool for screening SCAs.

To date, the assessment of SCAs in NB by NGS methods has yet to be validated. Additionally, the effectiveness of NGS compared with the conventional FISH has yet to be established. In this study, NGS analysis was used to detect SCAs in NB. Results of SCAs based on the two methods were compared to evaluate the possibility of using NGS as a complementary method to FISH.

## 2. Materials and Methods

### 2.1. Patients

This study retrospectively included 35 patients who were pathologically diagnosed with NB from January 2017 to December 2019 and analyzed using the FISH and the NGS. Excision was performed at diagnosis if the primary tumor was resectable. Otherwise, incisional biopsy was performed, followed by definitive surgery after neoadjuvant chemotherapy. Treatment was performed according to the risk group. Two alternating regimens of chemotherapy included CEDC (cisplatin, etoposide, doxorubicin, and cyclophosphamide) and ICE (ifosfamide, carboplatin, and etoposide), described previously [11]. Low-risk patients with stage 2 tumors received six cycles of chemotherapy; intermediate-risk patients received nine cycles of chemotherapy plus differentiation therapy (13-cis-retinoic acid) with or without local radiotherapy to the primary site. High-risk patients were treated with nine cycles of induction chemotherapy before tandem high-dose chemotherapy with autologous stem cell transplantation (HDCT/auto-SCT) [12]. Local radiotherapy and differentiating therapy with 13-cis retinoic acid were administered to all high-risk patients.

The tumor was classified based on the International Neuroblastoma Pathology Classification (INPC) [13], which subclassified tumors into favorable and unfavorable groups according to the histologic subtype of tumor, patient’s age, and MKI. The stage was assigned according to the international neuroblastoma staging system (INSS) [14]. The serum lactic dehydrogenase (LDH), ferritin, and neuron-specific enolase (NSE) levels, and urine vanillylmandelic acid (VMA) levels were measured during diagnostic work-up period.

### 2.2. FISH

Paraffin-embedded tissue obtained via biopsy or surgery at initial diagnosis was subjected to FISH, which tested 1p deletion, 11q deletion, and 17q gain. The hybridization studies were performed using the following probes: ZytoLight SPEC 1p36/1q25 Dual Color probe (Zytovision, Bremerhaven, Germany), Vysis LSI ATM (11q22) SpectrumOrange/CEP11 SpectrumGreen (Abbott Molecular, Abbott Park, IL, USA), and Vysis TOP2A (17q21)/CEP17 FISH (Abbott Molecular, Abbott Park, IL, USA). Each deletion of chromosome 1p and 11q was assigned to 1p36/1q25 and 11q22/CEP11 ratio lower than 0.67. A 17q21/CEP17 ratio higher than 1.3 defined the gain of chromosome 17q. The guidelines recommended by the INRG biology committee were used to define SCAs [15].

### 2.3. NGS

Targeted NGS was performed using CancerSCAN version 2 for 18 samples and PedSCAN for 17 samples. CancerSCAN version 2 and PedSCAN are hybridization capture-based NGS assays that detect single nucleotide variants (SNVs), small insertions and deletions, structural variants, copy number alterations, and microsatellite instability [16]. CancerSCAN version 2 is designed to detect mutations in 381 cancer-related genes. PedSCAN is designed to detect copy number alterations across the entire chromosomal region in addition to mutations in 353 genes related to pediatric cancers. The target genes of each assay are listed in Table S1. Genomic DNA isolation, library preparation, sequencing, and BAM file processing were performed as previously described [16].

Regarding SCA analysis, we used the DepthOfCoverage tool in GATK v3.1-1 to calculate the sequencing coverage for each target region. The mean coverage for each region was normalized by pattern matched normal reference datasets, and GC bias was corrected using LOESS regression. The log2 copy ratio of each region was adjusted for tumor purity which was calculated using B allele frequencies of heterozygous single nucleotide polymorphisms on the regions with one copy deletion, loss of heterozygosity, or one copy gain. The mean value of adjusted log2 copy ratio for each gene was plotted to manually measure the SCA of chromosomal arms based on the following criteria: (1) n case of diploid chromosomes where the log2 copy ratio of genes was evenly distributed around 0, the upper and lower reference lines were drawn so that 90% of log2 copy ratio of genes was within the two lines. (2) If the log2 copy ratio was not even across the chromosome arm, it was divided into segments with a thickness equal to that of diploid chromosomes. (3) If the center of the segment exceeded the reference line and the length of the segment was greater than one-third of the arm, it was defined as gain or deletion. (4) Whole chromosomal aberrations, which showed simultaneous and balanced alteration of p and q arms in a chromosome, were excluded from the SCA.

In the case of chromosomes 1, 11, and 17, we reconfirmed the proportion of SCA involved in the chromosomal arm by magnifying the copy number plots, which were adjusted according to the gene location in the NGS panel (Figure S1).

### 2.4. Statistical Analysis

McNemar test was performed to determine the difference between FISH and NGS for the detection of 1p deletion, 11q deletion, and 17q gain. Chi-square and Fisher’s exact tests were used to assess the correlation between the three SCAs and other clinical factors. Simple linear regression analysis was used to analyze the correlation of the copy number ratios of NGS with those of FISH. Mann–Whitney U tests were used to compare the copy numbers between the groups. *P*-values less than 0.05 were considered significant in all the analyses, and SPSS ver. 27.0 (IBM Corp., Chicago, IL, USA) was used.

## 3. Results

### 3.1. Clinicopathologic Features of the Patients

Table 1 and Supplementary Figure S2 show the clinicopathologic characteristics of all 35 patients diagnosed with NB. The median age of these patients was 22.3 months, and seven of these patients were aged ≤18 months and 28 were >18 months. Twenty-one of these patients were males and 14 were females. Histological classification of INPC revealed 14 cases of favorable histology and 21 cases of unfavorable histology. Furthermore, 22 patients were at stage 4. Six patients suffered from relapse, and only one of the relapsed patients died. Except for an intermediate-risk patient at stage 1, all patients underwent six or more cycles of chemotherapy.

### 3.2. NGS Analysis of SCAs of the Whole Chromosome Set

**Figure 1:**
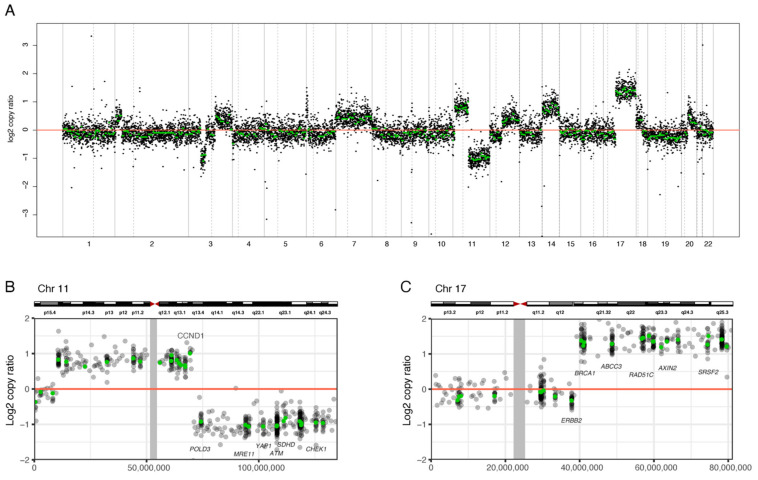
A representative copy number plot of NGS, showing 11q deletion, 17q gain, and no 1p deletion (Case No. 21). (**A**) Copy number plot for whole chromosome. Dashed line shows the location of the centromere. The exact position of the copy number change in chromosome 11 and chromosome 17 is shown in (**B**,**C**) respectively. Green dots represent the copy number of target genes. Gray line shows the location of the centromere.

The copy number plots of the whole set of chromosomes generated by NGS were manually reviewed to detect the SCAs (Supplementary Figure S3). The segmental gain was most common in 17q (14/35, 40%), followed by 2p (9/35, 25.7%), and 13q (7/35, 20%). The segmental deletion was most common in 11q (14/35, 40%), followed by 14q and 22q (9/35, 25.7%), and 1p (7/35, 20%). Figure 1 displays a representative copy number plot of the SCAs.

### 3.3. Comparison of SCAs by FISH and NGS

Table 2 shows the number of SCA-detected cases based on NGS compared with that based on FISH. McNemar test did not show any significant differences between FISH results and NGS results for 1p deletion, 11q deletion and 17q gain.

NGS revealed 1p deletion in 7 out of 35 cases (20%). NGS revealed the same results of 1p deletion as FISH in 33 out of the total 35 cases (94% concordance, *p* < 0.001; five cases of 1p deletion and 28 cases of no 1p deletion). Two cases were mismatched as deletion by NGS versus no deletion FISH. Among the two discrepant cases, the copy number ratio by FISH in one case was decreased to 0.75, which was not adequate based on the criteria (<0.67), whereas it was 1.05 in the other case.

**Figure 2:**
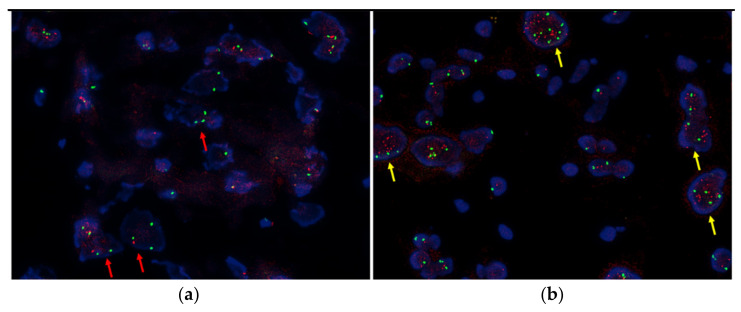
FISH detecting a few tumor cells with definite chromosomal alteration. (**a**) 11q deletion (red arrows). (**b**) 17q gain (yellow arrows).

The 11q deletion was detected by NGS in 13 out of 35 cases (37.1%). The results on 11q obtained via NGS were concordant with those acquired by FISH in 29 (8 cases of 11q deletion and 21 cases of no 11q deletion) out of 34 cases (85.3%, *p* < 0.001). FISH failed in one case, which revealed a 11q deletion via NGS. The discrepancy between NGS and FISH was found in five cases; the 11q deletion was detected only by NGS in four cases and only by FISH in one case. In all four cases where FISH missed detection of 11q deletion, copy number ratio was lowered to less than 0.8, but not lower than the diagnostic criteria (0.67). The microscopic section of the case where the detection of 11q deletion was missed by NGS contained large amounts contained large amounts of schwannian stromal components with a few neuroblasts. The 11q deletion was detected only in the neuroblastic cells by FISH (Case No. 35, Figure 2A).

NGS detected 17q gain in 15 out of 35 cases (42.9%). The NGS results of 17q were concordant with the FISH results in 25 out of 35 cases (71.4%, *p* = 0.01). 17q gain was detected by NGS but not by FISH in 9 cases. These cases showed a “subsegmental” 17q gain of NGS involving less than two-thirds of the long arm. In one case, the 17q gain was detected definitely in only a few cells by FISH (Case No. 23, Figure 2B) and NGS could not detect it.

We tested the association between clinical information and the SCAs. Unfavorable INPC histology was associated with 11q deletion detected by NGS (*p* = 0.022), 17q gain detected by NGS (*p* = 0.005), and 1p deletion detected by FISH (*p* = 0.049). The stage 4 disease was significantly associated with 1p deletion (*p* = 0.033) and 17q gain (*p* = 0.003) detected by NGS. The 17q gain identified by NGS was significantly associated with a high-risk group (*p* = 0.001). We could not show the association between the relapse of disease and SCAs. Three cases with MYCN amplification were included in this study; 1p deletion was detected in all of them, 11q deletion in none, and 17q gain in two of them. 1p deletion by FISH (*p* = 0.002) and by NGS (*p* = 0.005) were significantly correlated with MYCN amplification.

### 3.4. Copy Number Ratio of 1p, 11q, and 17q Measured by NGS

**Figure 3:**
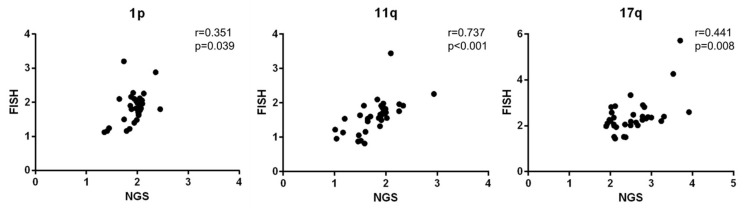
Copy numbers of 1p, 11q, and 17q were statistically correlated between NGS and FISH.

**Figure 4:**
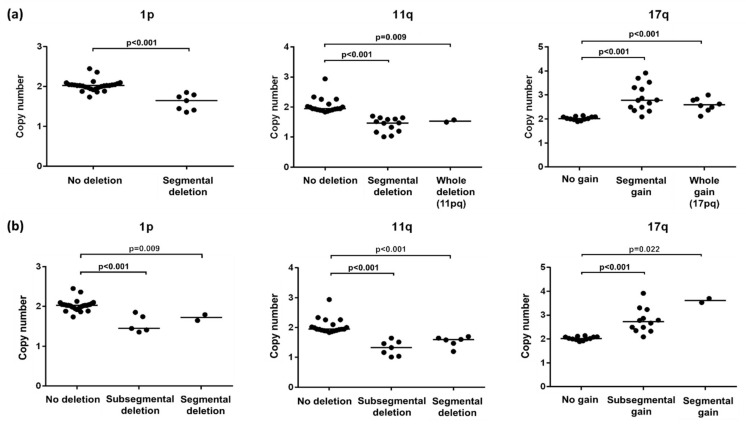
Comparison of copy numbers among SCA, no SCA, whole chromosome alteration (**a**), and among subsegmental, segmental chromosome alteration and whole chromosome alteration (**b**) by NGS.

The copy number ratios of 1p, 11q, and 17q calculated from NGS correlated with those obtained from FISH (*p* = 0.039, *p* < 0.001, and *p* = 0.008, respectively) (Figure 3). The copy number ratios of cases with SCA were clearly different from the copy number ratios of cases without SCA (1p, *p* < 0.001; 11q, *p* < 0.001; and 17q, *p* < 0.001). The copy number ratios of SCA did not differ significantly from that of whole chromosomal alterations nor “subsegmental” chromosomal alteration (Figure 4).

## 4. Discussion

NB originates from neural crest precursor cells of the developing sympathetic nervous system. It is notably heterogeneous with the clinical course and overall prognosis strongly correlates with their individual genetic or molecular status. SCA, one of the unbalanced chromosomal genetic alterations, has been associated with tumor aggressiveness, whereas the balanced alteration of a whole chromosome, known as numerical chromosomal alteration, was correlated with excellent overall survival and event-free survival [6,17]. A previous study reported that the SCAs 1p deletion, 11q deletion, and 17q gain were associated with worse prognostic parameters (aged >18 months and stage 4) [4]. Moreover, 11q deletion and 17q gain were correlated with poor survival outcome [4]. The deletion of 11q, usually inversely correlated with MYCN amplification was found in a quarter of patients with NB, aged mostly above 18 months at diagnosis [18]. Conversely, 1p deletion and 17q gain were associated with MYCN amplification [19,20]. In addition, the presence of 1p abnormalities in NB was associated with unresectable and metastatic tumors [21] and poor survival outcome [22]. The molecular risk stratification has been important for improving the prognosis of patients with NB [23]. In this study, we found that 1p deletion was statistically correlated with stage 4 by NGS and unfavorable INPC histology by FISH. Additionally, 11q deletion of NGS was significantly associated with unfavorable histology based on INPC. The NGS result of 17q gain was significantly associated with stage 4, a high-risk group, and unfavorable INPC histology. MYCN amplification was associated with 1p deletion. Even though the other SCAs were not correlated with MYCN amplification, 17q gain was detected in two out of three cases and 11q deletion was not detected in none of three cases, which showed the similar tendency to previous studies [18,19,20,24]. The discrepancy of statistical analysis would be due to the small number of total cases and that of the cases with MYCN amplification in this study.

FISH is one of the standard methods for the evaluation of chromosomal instability in NB. It allows to assess both numerical and structural chromosomal alterations and quantify the variations across the cell population [25]. However, this cytogenetic technique requires prior knowledge of the location of gene alteration to design specific probes. Each study requires detection of chromosomal aberrations using a limited number of DNA loci at one time. In addition, the squeezing tissue artifacts and technical errors related to the nuclear probe may yield misleading results [7].

Another applicable method is microarray comparative genomic hybridization (CGH). It is a genome-wide analysis to detect the structural alteration through the whole genomic DNA sequence that cannot be detected microscopically using FISH [26]. However, the disadvantage of array CGH is its inability to identify the specific gene alterations that do not result in copy number changes such as single nucleotide variants (SNVs) [27] and chromosomal rearrangements via conventional protocols [28].

Finally, the advent of a new technique, NGS, facilitates simultaneous detection of gene alteration, for example, copy number alterations, deletions, insertions, chromosomal rearrangements, and single nucleotide variants, in a cost- and tissue-efficient manner as a single test. It is no longer adequate to use single-gene assays such as FISH or genomic imbalance analysis in a whole genome, like CGH, exclusively. The NGS-based analysis has also increased the expectation to define the precise subgroup of cancers based on comprehensive genetic variations, thereby giving an opportunity for personalized therapy [29]. Further, NGS provides another tool for screening SCAs, by using the numerous probe sets based on whole chromosomes.

In particular, our panel of NGS also included probes along the whole chromosomes for accurate detection of SCAs. However, a comparison of FISH and NGS to determine the instability of 1p, 11q, and 17q in NB has previously not been investigated. Here, we compared the two approaches used to detect SCAs in NB. Our study showed that copy numbers of 1p deletion, 11q deletion, and 17q gain were statistically correlated with FISH and NGS. As stated previously, specific correlations between clinical information and SCAs of 1p, 11q, and 17q were confirmed by NGS. Interestingly, NGS revealed “subsegmental” gain, involving the variable length (at least one-third but less than two-thirds) of 17q, which was not detected by FISH. SCAs such as 11q deletion and 17 gain have been reported to show variable length; the shortest 11q deletion spanned from 11q23.1 to 11qter, involving about one-third of the long chromosomal arm [30]. Such “subsegmental” deletions of 11q cannot be detected by FISH, because 11q21 is the site of the representative detection probe. Moreover, patients with 17q gain involving less than 2/3 of the chromosomal arm, revealed poor outcome compared with those without chromosomal alteration [31]. Therefore, it is crucial to detect “subsegmental” alterations shorter than those detected via routine FISH methods, using techniques such as NGS. Unexpectedly, there was no significant difference in the copy number between the “subsegmental” gain/deletion and segmental gain/deletion by NGS, in spite of the algorithm used to calculate for the whole short (or long) arm, in contrast to the significant difference in copy number between SCAs and absence of chromosomal alteration. The limited sample size and the heterogeneous tumor purity of each case would explain the lack of difference in copy numbers between cases of “subsegmental” and segmental alteration. The limited sample size underscores the need for further validation of the results and investigation of the significance of “subsegmental” SCA using larger samples.

Some of cases with SCA detected only by NGS showed the decreased or increased copy number ratio by FISH as well, which did not meet the diagnostic criteria. It might be partly related to the use of the 2-dimensional sections from paraffin embedded tissue instead of whole-cell preparation for FISH in this study. The copy number ratio in this situation would be affected by variation of the nuclear size of tumor cells, tumor cell density, section thickness, and so on.

On the other hand, NGS was barely able to detect SCAs in a few blast cells with abundant and differentiated stroma. In this study, an 11q deletion (case No. 35) and a 17q gain (case No. 23) could be detected by FISH (Figure 2), but not by NGS. When the microscopic section reveals only a small number of neuroblasts in the background of abundant schwannian stroma, FISH is more reliable than NGS, and the combination of FISH with NGS is recommended.

Another limitation of this study was that the association of SCAs with overall survival could not be established due to the short follow-up period when only one patient died. A large study for longer period is required to determine the clinical significance.

## 5. Conclusions

In conclusion, NGS with a copy number plot was comparable to FISH for SCA detection and increased sensitivity to detect “subsegmental” alteration. NGS is recommended for detecting SCAs as a complementary method. Further, it can be a cost-effective and tissue-efficient alternative analysis to the conventional FISH for detecting comprehensive molecular alterations.

## Figures and Tables

**Table 1:** Summarization of the clinicopathologic information in patients of neuroblastoma.

	Number (*n* = 35)	Median (Range)
Age (years)		3.08 (11.2–0.2)
Sex		
Male	21	
Female	14	
Stage		
1	2	
2	6	
3	4	
4	23	
MYCN status		
Amplification	3	
No amplification	32	
Risk group		
High risk	22	
Non high risk	13	
Primary site		
Abdomen	27	
Mediastinum	8	
Diagnosis		
Neuroblastoma, differentiating	7	
Neuroblastoma, poorly differentiated	17	
Neuroblastoma, undifferentiated	1	
Ganglioneuroblastoma	6	
Ganglioneuroblastoma, intermixed type (GNB-I)	3	
Ganglioneuroblastoma, nodular type (GNB-N)	1	
INPC		
Favorable	21	
Non-favorable	14	
LDH (IU/L)		590 (239–7789)
Ferritin (ng/mL)		93.3 (8.3–3771.8)
NSE (ng/mL)		46.8 (10.1–685)
VMA (mg/day)		6.9 (0.4–588.4)
Relapse		
Yes	6	
No	29	
Death		
Yes	1	
No	34	

**Table 2:** Assignment of segmental chromosomal aberrations: NGS versus FISH.

1p Deletion			FISH				Total
		Deletion	(%)	No Deletion	(%)		(%)
NGS	Deletion	5	(71.4)	2	(28.6)		7
	(%)	(100)		(6.7)			(20)
	No deletion	0	(0)	28	(100)		28
	(%)	(0)		(93.3)			(80)
Total (%)		5 (14.3)		30 (85.7)			35 (100)
**11q deletion**			**FISH**				**Total**
		Deletion	(%)	No deletion	(%)	Fail	(%)
NGS	Deletion	8	(61.5)	4	(30.8)	1	13
	(%)	(88.9)		(16)			(37.1)
	No deletion	1	(4.8)	21	(95.2)		22
	(%)	(11.1)		(84)			(62.9)
Total (%)		9 (25.7)		25 (71.4)		1 (2.9)	35 (100)
**17q gain**		**FISH**					**Total**
		Gain	(%)	No gain	(%)		(%)
NGS	Gain	6	(40)	9	(60)		15
	(%)	(85.7)		(32.1)			(42.9)
	No gain	1	(5)	19	(95)		20
	(%)	(14.3)		(67.9)			(57.1)
Total (%)		7 (20)		28 (80)			35 (100)

## Data Availability

The data presented in this study are available on request from the corresponding author. The data are not publicly available due to the privacy of the patients.

## Supplementary Materials

The following are available online at https://www.mdpi.com/article/10.3390/diagnostics11091702/s1, Figure S1. An adjustment of gene location in the redesigned copy number plots, showing 11q deletion, 17q gain, and no 1p deletion (Case No. 21), Figure S2. Clinicopathological information of 35 neuroblastoma cases, Figure S3. SCAs in the whole chromosomes of 35 neuroblastoma cases, Table S1. Target gene list of NGS assays.
