# Supplementary material for: Comparison of Next-Generation Sequencing and Fluorescence In Situ Hybridization for Detection of Segmental Chromosomal Aberrations in Neuroblastoma
Source: Diagnostics (Basel). 2021 Sep 17;11(9):1702. doi: 10.3390/diagnostics11091702 (PMC8465051; doi:10.3390/diagnostics11091702)

Figure S1. An adjustment of gene location in the redesigned copy number plots, showing 11q deletion, 17q gain, and no 1p deletion (Case No. 21).

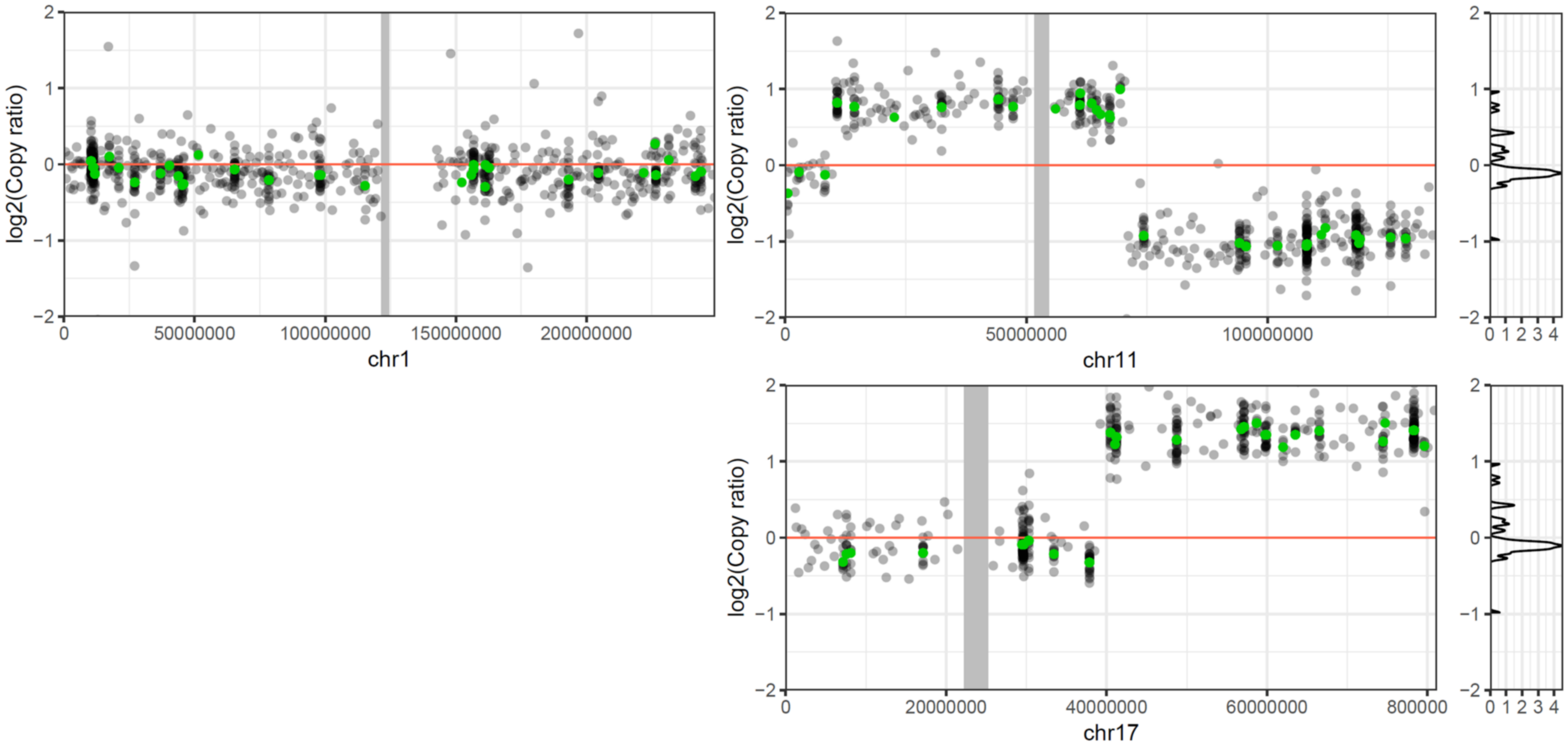

Figure S2. Clinicopathological information of 35 neuroblastoma cases.

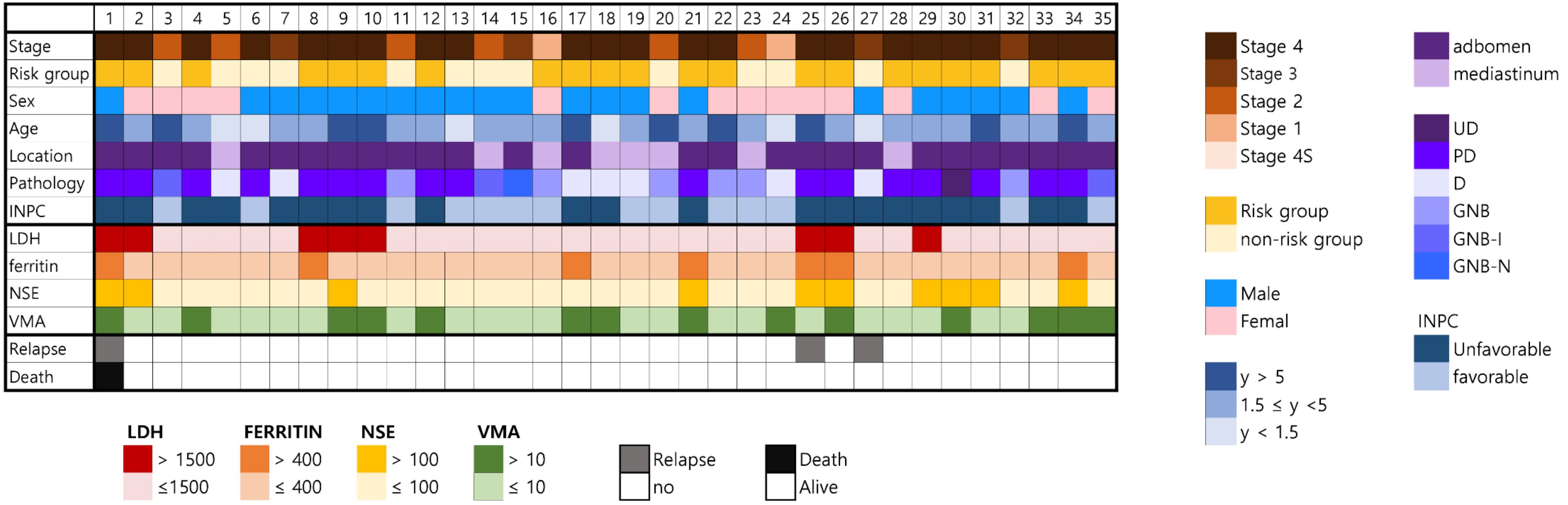

Figure S3. SCAs in the whole chromosomes of 35 neuroblastoma cases.

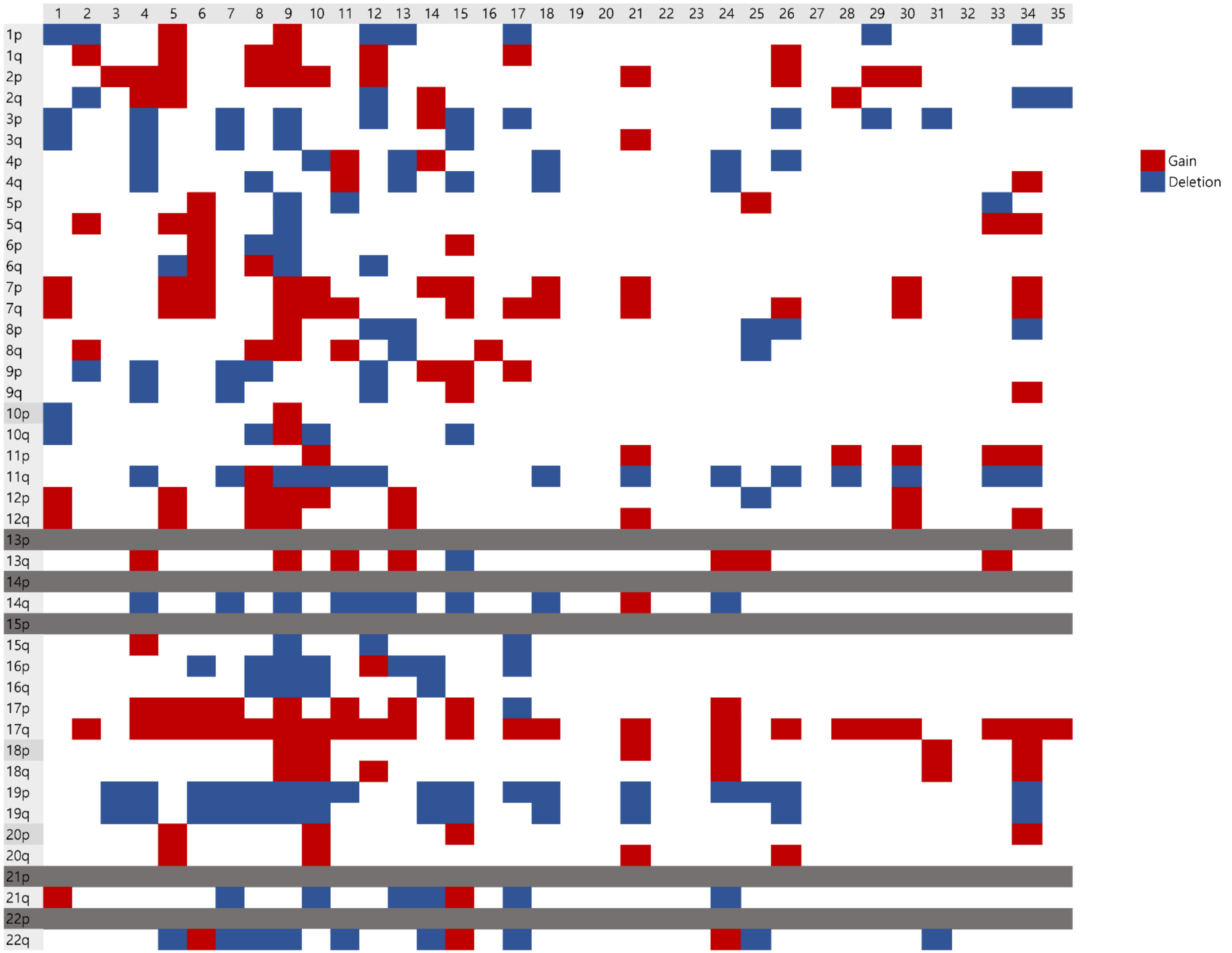

Supplement: Supplementary file 1 [file diagnostics-11-01702-s001.zip › diagnostics-1357025 Figures S1-3.pdf]
