# Supplementary material for: Comparison of Next-Generation Sequencing and Fluorescence In Situ Hybridization for Detection of Segmental Chromosomal Aberrations in Neuroblastoma
Source: Diagnostics (Basel). 2021 Sep 17;11(9):1702. doi: 10.3390/diagnostics11091702 (PMC8465051; doi:10.3390/diagnostics11091702)
Supplement: Supplementary file 1 [file diagnostics-11-01702-s001.zip › diagnostics-1357025 Table S1.pdf]

Table S1. Target gene list of NGS assays

CancerSCAN version 2								PedSCAN							
ABL1	CARD11	DOCK2	FGFR4	KIT	NTRK3*	REL	TMPRSS2	ABCB1	CBL	ERBB3	GLI2	MPL	PIK3CB	SEMA3C	WRN
ABL2	CASP8	DOT1L	FLT1	KLF4	NUP93	RET*	TNFAIP3	ABCB11	CBLB	ERBB4	GNA11	MRE11A	PIK3R1	SERPINB3	WT1
ACVR1B	CBFB	EGFR	FLT3	KLHL6	PAK3	RICTOR	TNFRSF14	ABCC1	CCND1	ERCC1	GNAQ	MSH2	PIK3R2	SETBP1	XPA
AKT1	CBL	ELMO1	FLT4	KRAS	PAK7	RNF43	TNKS	ABCC3	CCND2	ERCC2	GNAS	MSH3	PINK1	SETD2	XPC
AKT2	CCND1	EP300	FOXA1	LMO1	PALB2	ROBO1	TNKS2	ABCG2	CCND3	ERCC3	GPC3	MSH6	PML	SF3B1	XRCC1
AKT3	CCND2	EPHA3	FOXL2	LRP1B	PARP1	ROBO2	TOP1	ABL1	CD79A	ERCC4	GPC4	MTHFR	PMS1	SH2B3	XRCC3
ALK*	CCND3	EPHA5	FUBP1	LRP6	PARP2	ROS1*	TP53	ACVR1	CD79B	ERCC5	GREM1	MTOR	PMS2	SHH	YAP1
ALOX12B	CCNE1	EPHA6	GATA1	LTK	PARP3	RPA1	TRAF7	AHNAK2	CDC73	ERCC6	GSTM1	MTRR	POLD1	SHOC2	ZNRF3
APC	CD79A	EPHA7	GATA2	MAML1	PARP4	RPTOR	TRRAP	AIP	CDH1	ERG	GSTP1	MUC16	POLD3	SHROOM2	
APCDD1	CD79B	EPHB1	GATA3	MAP2K1	PAX5	RUNX1	TSC1	AKT1	CDK4	ESR1	H3F3A	MUC4	POLE	SLCO1B1	
APOBEC3A	CDC42	EPHB4	GID4	MAP2K2	PBRM1	RUNX1T1	TSC2	AKT2	CDK6	ETV6	HDAC2	MUTYH	POT1	SMAD4	
APOBEC3B	CDC73	EPHB6	GNA11	MAP2K4	PDGFRA	SEMA3A	TSHR	AKT3	CDK8	EWSR1*	HIF1A	MYB	POU6F2	SMAD7	
AR	CDH1	ERBB2	GNA13	MAP3K1	PDGFRB	SEMA3E	U2AF1	ALK*	CDKN1C	EXT1	HIST1H3B	MYC	PPM1D	SMARCA1	
ARAF	CDH2	ERBB3	GNAQ	MAP3K13	PDK1	SETBP1	U2AF2	AMER1	CDKN2A	EXT2	HMBS	MYCL	PPOX	SMARCA4	
ARFRP1	CDH20	ERBB4	GNAS	MCL1	PGR	SETD2	USP9X	ANKRD26	CDKN2B	EZH2	HNF1A	MYCN	PRCC	SMARCB1	
ARID1A	CDH5	ERCC2	ADGRA2	MDM2	PHF6	SF3A1	VHL	APC	CDKN2C	FAH	HRAS	MYD88	PRF1	SMO	
ARID1B	CDK12	ERG	GRIN2A	MDM4	PHLPP2	SF3B1	WHSC1L1	APOBEC3A	CEBPA	FANCA	IDH1	NBN	PRKAR1A	SNCAIP	
ARID2	CDK4	ESR1	GSK3B	MED12	PIK3C3	SRSF7	WISP3	APOBEC3B	CEP57	FANCB	IDH2	NF1	PTCH1	SOD2	
ASXL1	CDK6	ETV1	GUCY1A2	MEF2B	PIK3CA	SH2B3	WT1	ARID1A	CHEK1	FANCC	IDO1	NF2	PTCH2	SOS1	
ATM	CDK8	ETV4	H3F3A	MEN1	PIK3CG	SKP2	WWP1	ARID1B	CHEK2	FANCD2	IGF1	NFE2L2	PTEN	SPRED1	
ATR	CDKN2A	ETV5	HGF	MET	PIK3R1	SLIT2	XBP1	ARID2	CIC	FANCE	IGF1R	NFKBIA	PTPN11	SPRTN	
ATRX	CDKN2B	ETV6	HIST1H3B	MITF	PIK3R2	SMAD2	XPO1	ASXL1	COLCA2	FANCF	IGF2R	NHP2	PTPRD	SRC	
AURKA	CDKN2C	EWSR1*	HNF1A	MLH1	PKHD1	SMAD3	XRCC3	ATIC	CREBBP	FANCG	IKZF1	NKX2-1	RAB27A	SRSF2	
AURKB	CDX2	EYA2	HOXA3	MPL	PLCG1	SMAD4	ZNF217	ATM	CSF1R	FANCI	IL7R	NOP10	RAC1	SS18**	
AXIN1	CEBPA	EZH2	HRAS	MRE11A	PMS2	SMARCA1	ZNF703	ATR	CSF3R	FANCL	ITK	NOTCH1	RAD21	STAG2	
AXL	CHD1	AMER1	HSP90AA1	MSH2	PNRC1	SMARCA4	ZRSR2	ATRX	CSMD1	FANCM	JAK1	NOTCH3	RAD50	STAT3	
B2M	CHD2	FAM46C	IDH1	MSH6	PPP2R1A	SMARCB1		AURKA	CTDNEP1	FAT1	JAK2	NPAT	RAD51C	STK11	
BACH1	CHD4	FANCA	IDH2	MTOR	PRDM1	SMARCD1		AURKB	CTNNB1	FBXW7	JAK3	NPM1	RAD51D	SUFU	
BAP1	CHEK1	FANCC	IGF1	MUTYH	PRKAR1A	SMO		AXIN1	CTR9	FGFR1	KDM6A	NRAS	RAF1	SUZ12	
BARD1	CHEK2	FANCD2	IGF1R	MYC	PRKDC	SOCS1		AXIN2	CYLD	FGFR2	KDR	NSD1	RB1	TCF12	
BCL2	CHUK	FANCE	IGF2R	MYCL	PRPF40B	SOX10		B2M	DDB1	FGFR3	KIF1B	NT5C2	RECQL4	TERT	
BCL2A1	CIC	FANCF	IKBKE	MYCN	PRSS8	SOX2		BAP1	DDB2	FGFR4	KIT	NTHL1	RELA**	TET2	
BCL2L1	CRBN	FANCG	IKZF1	MYD88	PTCH1	SOX9		BARD1	DDR2	FH	KMT2A	NTRK1	REST	TFE3	
BCL2L2	CREBBP	FANCI	IL7R	NCOA3	PTCH2	SPEN		BCL2	DDX3X	FLCN	KMT2C	NTRK2	RET*	TGFBR1	
BCL6	CRKL	FANCL	INHBA	NCOR1	PTEN	SPOP		BCL6	DICER1	FLG	KMT2D	NTRK3	RHBDF2	TGFBR2	
BCOR	CRLF2	FANCM	INPP4B	NF1	PTPN11	SRC		BCOR	DIS3L2	FLI1	KRAS	NUDT15	RHPN2	TINF2	
BCORL1	CRLF2	FAT3	INSR	NF2	PTPRD	SRSF1		BCORL1	DNMT3A	FLT3	LAMA5	ODZ3	RICTOR	TMEM127	
BCR	CSF1R	FBXW7	IRF4	NFE2L2	RAB35	SRSF2		BLM	DPYD	FOXL2	LMO1	OR5T1	RIT1	TP53	
BLM	CTCF	FGF10	IRS2	NFKBIA	RAC1	STAG2		BMP4	DROSHA	FOXO1**	LRP1B	OTX2	RNF213	TPMT	
BRAF	CTNNA1	FGF12	ITK	NKX2-1	RAC2	STAT3		BMPR1A	DUSP10	FOXR2	MAP2K1	PALB2	ROS1*	TRIM37	
BRCA1	CTNNB1	FGF14	JAK1	NKX3-1	RAD50	STAT4		BRAF	EBF1	FRS2	MAP2K2	PARP1	RRAS2	TSC1	
BRCA2	CUL4A	FGF19	JAK2	NOTCH1	RAD51	STK11		BRCA1	EGFR	FUBP1	MAX	PAX5	RTEL1	TSC2	
BRD2	CUL4B	FGF23	JAK3	NOTCH2	RAD51B	SUFU		BRCA2	EIF3H	G6PC	MDM2	PBRM1	RUNX1	TTN	
BRD3	CYLD	FGF3	JUN	NOTCH3	RAD51C	SYK		BRIP1	EML4	G6PD	MDM4	PDE6G	RYR1	TXNDC15	
BRD4	CYP17A1	FGF4	KAT6A	NOTCH4	RAD51D	TBX22		BTK	EP300	GAB1	MED12	PDGFRA	SBDS	TXNDC16	
BRIP1	DAXX	FGF6	KDM5A	NPM1	RAD52	TBX3		BUB1B	EPCAM	GATA1	MEN1	PDGFRB	SDHA	TYK2	
BTG1	DDR2	FGF7	KDM5C	NRAS	RAD54L	TERT		C11ORF95	EPHA3	GATA2	MET*	PDPK1	SDHAF2	U2AF1	
BTK	DIS3	FGFR1*	KDM6A	NSD1	RAF1	TET2		C8ORF34	EPHB1	GATA3	MITF	PHF6	SDHB	UGT1A1	
EMSY	DNMT1	FGFR2*	KDR	NTRK1*	RARA	TGFBR2		CALR	EPHB4	GFI1B	MLH1	PHOX2B	SDHC	VHL	
NUTM1	DNMT3A	FGFR3*	KEAP1	NTRK2*	RB1	TIPARP		CARD11	ERBB2	GLI1	MN1	PIK3CA	SDHD	WAS	

* target region includes intronic fusion breakpoints

** target region covers fusion breakpoints only
